# A Comparative Study of Transurethral Resection of the Prostate (TURP) Versus Transurethral Incision of the Prostate (TUIP) in Small-Sized Prostate Glands With Benign Prostatic Hyperplasia

**DOI:** 10.7759/cureus.86848

**Published:** 2025-06-27

**Authors:** Mushtaq Hussain, Kanwal Naz, Ghulam Mustafa, Faisal Haneef, Tanzeel Gazder, Ahmad Waleed, Umair Zafar

**Affiliations:** 1 Urology, Sindh Institute of Urology and Transplantation, Karachi, PAK; 2 Urology, Queen Elizabeth Hospital Birmingham, Birmingham, GBR; 3 Urology, Sindh Institute of Urology and Transplantation, karachi, PAK; 4 Urology, Recep Tayyip Erdogan Hospital, Muzaffargarh, PAK

**Keywords:** benign prostatic hyperplasia, international prostate symptoms score (ipss), lower urinary tract symptoms, retrograde ejaculation, small prostate volume, transurethral incision of the prostate (tuip), transurethral resection of the prostate (turp)

## Abstract

Introduction and aim: Benign prostatic hyperplasia (BPH) is a prevalent condition in aging males, often leading to lower urinary tract symptoms (LUTS). While transurethral resection of the prostate (TURP) is the gold standard for surgical management, transurethral incision of the prostate (TUIP) is a less invasive alternative for small prostate volumes. This study aims to compare the efficacy and safety of TURP and TUIP in patients with small prostate glands (<30 mL) suffering from BPH.

Methods: A prospective cohort study was conducted from August 2024 to January 2025 on 70 patients divided into two groups: Group I (TURP, n = 35) and Group II (TUIP, n = 35) at the Urology Department of Sindh Institute of Urology and Transplantation, Karachi. Preoperative and postoperative parameters, including International Prostate Symptom Score (IPSS), urinary flow rates, voided volume, post-void residual (PVR), operative time, catheterization duration, and hospital stay, were analyzed. Statistical analysis was performed using IBM SPSS Statistics for Windows version 25.

Results: Both groups showed significant improvements in IPSS, average flow rate, maximum flow rate, and PVR at six-month follow-up. TURP had a longer operative time (mean: 61.2 min) compared to TUIP (mean: 32.4 min, p < 0.001). Catheterization duration and hospital stay were shorter in the TUIP group (p < 0.05). Blood transfusion was required in 5.7% of TURP patients and none in the TUIP group. Retrograde ejaculation was more common in the TURP group, 40% versus 12% in the TUIP group. No significant difference was noted in reoperation rates.

Conclusion: TUIP is a safe and effective alternative to TURP for small-sized prostates, offering comparable symptomatic relief with shorter operative times, reduced catheterization duration, and fewer complications.

## Introduction

Benign prostatic hyperplasia (BPH) is a histopathological condition characterized by the nonmalignant proliferation of epithelial and stromal components of the prostate gland [1]. It is a highly prevalent condition in aging men, with approximately 50% of men over 60 years experiencing symptoms, and nearly 25% eventually requiring surgical intervention [2,3]. BPH is a leading cause of lower urinary tract symptoms (LUTS), which include urinary frequency, urgency, hesitancy, decreased urinary stream, and nocturia [4].

The pathophysiology of BPH involves hormonal changes, chronic inflammation, and growth factor imbalances that result in prostatic enlargement and subsequent bladder outlet obstruction (BOO) [1]. LUTS significantly impacts the quality of life. Although medical therapy is usually the first-line approach, many patients fail to respond adequately or experience intolerable side effects, thereby requiring surgical intervention [5]. Even small prostates can cause significant bladder outlet obstruction, as shown by urodynamic studies [6].

Transurethral resection of the prostate (TURP) has long been considered the gold standard for the surgical management of BPH due to its effectiveness in relieving obstruction and improving urinary flow. However, TURP is associated with complications, including bleeding, TUR syndrome, erectile dysfunction, and retrograde ejaculation [5,7]. In patients with small-volume prostates (<30 mL), the risks of TURP may outweigh its benefits, particularly due to an increased risk of bladder neck contracture (BNC) [8].

Transurethral incision of the prostate (TUIP), a simpler and less invasive alternative, offers several advantages in appropriately selected patients. These include reduced operative time, shorter hospital stay, lower perioperative morbidity, and better preservation of antegrade ejaculation [4,9,10]. Despite guideline recommendations from American, Canadian, and European urological associations favoring TUIP in prostates <30 mL without a median lobe [4,9], the procedure remains underutilized due to concerns regarding long-term efficacy and subjective surgeon preference for TURP. This is largely attributed to concerns regarding long-term efficacy and a persistent surgeon preference for TURP [3].

This study aims to bridge the knowledge gap by prospectively comparing the outcomes of TURP and TUIP in patients with small-volume prostates to evaluate their relative efficacy and safety.

## Materials and methods

This prospective cohort study was conducted in the Department of Urology at the Sindh Institute of Urology and Transplantation (SIUT), Karachi, between August 2024 and January 2025, with follow-up to date. A total of 70 male patients were enrolled using a non-probability, consecutive sampling technique. The sample size was calculated using G*Power version 3.1.9.7 (Heinrich Heine University Düsseldorf, Germany), targeting a two-tailed t-test comparison of means with an effect size (Cohen’s d) of 0.6, alpha = 0.05, and statistical power of 80%. This yielded a minimum required sample of 30 patients per group. We included 35 patients per group to account for potential dropouts and ensure adequate statistical power to detect meaningful differences in the International Prostate Symptom Score (IPSS) and flow parameters between the TURP and TUIP groups.

Eligible participants were men aged over 45 years presenting with LUTS consistent with BPH. Inclusion criteria included a prostate volume of less than 30 mL confirmed by transrectal ultrasound (TRUS), an IPSS greater than 21, a maximum urinary flow rate (Qmax) below 10 mL/s, and failure of prior medical therapy. Patients with urethral stricture, enlarged median lobe, neurogenic bladder, active urinary tract infection, suspected malignancy (elevated prostate-specific antigen (PSA) or abnormal digital rectal examination (DRE)), or previous prostate or urethral surgery were excluded.

Patients were assigned to one of two groups. Group I underwent TURP, in which a 26 French resectoscope was used to resect the prostatic adenoma circumferentially from the bladder neck to the verumontanum, down to the prostatic capsule. Group II underwent TUIP, a less invasive procedure performed using a Collin’s knife to make bilateral incisions at the 5 and 7 o’clock positions from the bladder neck to the verumontanum, thereby relieving bladder outlet obstruction without removing tissue.

All procedures were performed under spinal anesthesia by a team of experienced urologists under the supervision of a senior consultant. Preoperative assessments included IPSS, uroflowmetry (Qmax, average flow, voided volume), and PVR urine measured via ultrasound. Operative parameters, including duration of surgery, intraoperative complications, catheterization time, and hospital stay, were recorded. Postoperative assessments were conducted at six months and included repeat IPSS scoring, uroflowmetry, PVR measurement, and evaluation for retrograde ejaculation and need for reoperation.

Data were analyzed using IBM SPSS Statistics for Windows, version 25.0 (released 2017, IBM Corp., Armonk, NY). Paired sample t-tests were employed to evaluate changes within each group. Independent t-tests and chi-square tests were used for between-group comparisons. A p-value of <0.05 was considered statistically significant.

## Results

Demographic and preoperative characteristics

Both groups were comparable in terms of baseline demographics and symptom severity (Table 1). The mean age in Group I (TURP) was 62.3 years, while Group II (TUIP) had a mean age of 61.4 years. IPSS values were also similar between groups, averaging 22.7 in Group I and 23.1 in Group II. These findings indicate no statistically significant differences in baseline characteristics.

**Table 1:** Demographic and baseline characteristics Data are presented as mean ± standard deviation (SD) unless otherwise indicated. Independent t-test applied for continuous variables. TURP: transurethral resection of the prostate, TUIP: transurethral incision of the prostate, PSA: prostate-specific antigen

Parameter	Group I (TURP)	Group II (TUIP)	Test statistic	p-value
Age (years)	62.3 ± 6.4	61.4 ± 5.9	t = 0.61	0.54
Prostate volume (mL)	28.2 ± 1.6	27.9 ± 1.8	t = 0.72	0.47
PSA (ng/mL)	1.9 ± 0.6	1.8 ± 0.5	t = 0.81	0.42
Diabetes mellitus (%)	9 (25.7%)	10 (28.6%)	χ² = 0.06	0.8
Preoperative IPSS	22.7 ± 2.1	23.1 ± 1.9	t = 0.81	0.42
Qmax (mL/s)	7.2 ± 1.5	7.3 ± 1.3	t = 0.29	0.77

Operative and postoperative outcomes

Operative parameters varied significantly between the groups (Table 2). TURP procedures had a longer mean operative time of 60.2 minutes compared to 31.5 minutes in the TUIP group. Similarly, catheterization duration was longer in the TURP group, averaging 3.1 days, whereas patients in the TUIP group required a mean of 1.7 days. Hospital stay was consistent across both groups, with a mean duration of one day. Blood transfusion was required in one patient (2.9%) in the TURP group, while no transfusions were needed in the TUIP group.

**Table 2:** Operative and postoperative outcomes Data are presented as mean ± standard deviation (SD). Independent t-tests were used for continuous variables, and a chi-square test was used for categorical variables. TURP: transurethral resection of the prostate, TUIP: transurethral incision of the prostate

Parameter	Group I (TURP) (n = 35)	Group II (TUIP) (n = 35)	Test statistic (t/χ²)	p-value
Operative time (min)	60.2 ± 6.8	31.5 ± 5.9	t = 19.24	<0.001
Catheter days	3.1 ± 0.7	1.7 ± 0.6	t = 9.67	<0.001
Hospital stay (days)	1 ± 0	1 ± 0	t = 0.00	1
Blood transfusion (%)	2.9% (1/35)	0% (0/35)	χ² = 1.02	0.31

Functional outcomes

Both surgical groups demonstrated substantial postoperative improvements in urinary symptoms and flow dynamics at the six-month follow-up (Table 3). In Group I, the mean IPSS improved from 22.7 to 11.9, while in Group II, it decreased from 23.1 to 12.2. The average urinary flow rate increased from 7.2 mL/s to 12.6 mL/s in the TURP group and from 7.3 mL/s to 12.4 mL/s in the TUIP group. The maximum flow rate also improved comparably, rising from 12.8 mL/s to 17.4 mL/s in Group I and from 13.1 mL/s to 17.5 mL/s in Group II. Post-void residual urine volume showed a marked reduction in both groups, with Group I decreasing from 89.1 mL to 23.5 mL and Group II from 82.4 mL to 25.2 mL.

**Table 3:** Functional outcomes Functional outcomes at the six-month follow-up. Values are mean ± SD. Paired t-tests were used for within-group comparisons; independent t-tests for between-group comparisons. TURP: transurethral resection of the prostate, TUIP: transurethral incision of the prostate, IPSS: International Prostate Symptom Score

Parameter	Group I (TURP)		Group II (TUIP)		t-value (between Δ)	p-value
	Preoperative	Postoperative	Preoperative	Postoperative		
IPSS	22.7 ± 2.1	11.9 ± 1.7	23.1 ± 1.9	12.2 ± 1.5	t = 0.75	0.45
Average flow rate (mL/s)	7.2 ± 1.5	12.6 ± 2.0	7.3 ± 1.3	12.4 ± 1.8	t = 0.39	0.69
Maximum flow rate (mL/s)	12.8 ± 2.3	17.4 ± 2.5	13.1 ± 2.2	17.5 ± 2.3	t = 0.18	0.86
Post-void residual (mL)	89.1 ± 14.3	23.5 ± 6.2	82.4 ± 13.1	25.2 ± 5.8	t = 1.21	0.23

Complications

Postoperative complications were minimal in both groups (Table 4). Retrograde ejaculation was reported in 40% of patients in the TURP group, significantly higher than the 12% observed in the TUIP group. No major adverse events were recorded in either group.

**Table 4:** Postoperative complications Comparison of postoperative complications between TURP and TUIP groups. Values shown as percentages. Chi-square test used for comparison. TURP: transurethral resection of the prostate, TUIP: transurethral incision of the prostate

Complication	Group I (TURP) (%)	Group II (TUIP) (%)	χ²-value	p-value
Retrograde ejaculation	40.0% (14/35)	12.0% (4/35)	χ² = 7.39	0.006
Blood transfusion	2.9% (1/35)	0.0% (0/35)	χ² = 1.02	0.31

## Discussion

This prospective cohort study demonstrates that both TURP and TUIP are effective surgical options for relieving LUTS in men with small-volume BPH. However, our findings indicate that TUIP offers several advantages over TURP in appropriately selected patients, including shorter operative time, reduced hospital stay, lower complication rates, and better preservation of sexual function. The improvements in IPSS, maximum urinary flow rate (Qmax), and PVR at six months were statistically significant and comparable between both surgical groups. These results align with existing literature demonstrating that TUIP provides symptomatic relief equivalent to TURP in men with prostate volumes <30 mL [5,11]. Moreover, TUIP is technically less complex and requires less operative time, making it a valuable option, particularly in settings with limited surgical resources or high patient volume. One of the most compelling findings from our study was the lower incidence of retrograde ejaculation among TUIP patients. This complication, frequently associated with TURP, is known to impact patient satisfaction and quality of life postoperatively [9,10]. The reduced rate of retrograde ejaculation in TUIP patients aligns with findings by Abd-El Kader et al. and Hueber et al. [3,10]. The anatomical rationale lies in the minimally invasive nature of TUIP, which preserves the ejaculatory ducts and neurovascular bundles by avoiding extensive resection and thermal injury to the prostatic tissue [8].

Despite these advantages, TUIP remains underutilized in clinical practice. Several factors may contribute to this trend, including a longstanding preference for TURP as the surgical gold standard, variability in surgical training, and limited awareness of current guideline recommendations. The American Urological Association (AUA), Canadian Urological Association (CUA), and European Association of Urology (EAU) all endorse TUIP as a first-line option in patients with prostate volumes under 30 mL and without a prominent median lobe [4]. Broader dissemination and adoption of these recommendations are needed to improve guideline-concordant care.

Concerns have been raised regarding the long-term durability of TUIP and the potential for reintervention. However, several studies suggest that when performed in properly selected patients, TUIP has low reoperation rates and favorable long-term outcomes [9]. Further large-scale, randomized trials with extended follow-up are warranted to reinforce the evidence base and address residual concerns regarding durability.

Limitations

This study has limitations. It was conducted at a single tertiary care center, which may limit generalizability. In addition, the follow-up period of six months, while adequate to assess short-term outcomes, does not capture long-term efficacy or late-onset complications. Future research should include longer follow-up durations, patient-reported outcome measures, and health economic evaluations to compare cost-effectiveness.

## Conclusions

This study demonstrates that both TURP and TUIP significantly improve urinary symptoms and flow metrics in men with small-volume BPH. However, TUIP offers several practical advantages, including reduced operative time, shorter catheterization, fewer postoperative complications, and lower rates of retrograde ejaculation. These findings support the consideration of TUIP as a first-line surgical intervention in appropriately selected patients, particularly those prioritizing preservation of sexual function. These findings reinforce guideline recommendations supporting TUIP for appropriately selected patients and advocate for its broader adoption, especially in high-volume or resource-limited settings. However, conclusions are limited to early postoperative outcomes, and future studies with longer follow-up are needed to further assess the long-term durability and patient satisfaction associated with both procedures.
